# Advances in biosensor technologies for the detection of antimicrobial resistance in *Staphylococcus aureus*

**DOI:** 10.3389/fcimb.2025.1741845

**Published:** 2026-01-09

**Authors:** Mario Pérez-Rodríguez, Esther Serrano-Pertierra, María Carmen Blanco-López

**Affiliations:** 1 Department of Physical and Analytical Chemistry, University of Oviedo, Oviedo, Spain; 2 Department of Biochemistry and Molecular Biology, University of Oviedo, Oviedo, Spain

**Keywords:** antimicrobial resistance, biosensors, methicillin-resistant *Staphylococcus aureus* (MRSA), point-of-care diagnostics, rapid pathogen detection

## Abstract

The rise of methicillin-resistant *Staphylococcus aureus* (MRSA) underscores the urgent need for rapid, sensitive, and portable diagnostics. In this paper, we have critically reviewed recent advances in biosensor technologies, integrating nanomaterials, aptamers, CRISPR/Cas systems, and microfluidic lab-on-a-chip platforms, that enable sub-hour and ultrasensitive detection of *S. aureus* and its resistance genes. These innovations offer powerful alternatives to conventional culture and PCR assays, forming the way for real-time, point-of-care antimicrobial resistance testing. Remaining challenges include matrix interference, lack of standardization, and limited clinical validation, yet continued integration with artificial intelligence and digital systems promises transformative diagnostic capabilities.

## 1 Introduction

Multidrug-resistant (MDR) pathogens, particularly *Staphylococcus aureus* and its methicillin-resistant variants (MRSA), pose a significant and continually evolving threat to global public health. This challenge arises from the convergence of extensive antimicrobial resistance with a diverse array of virulence factors. Clinically, *S. aureus* is responsible for a broad spectrum of infections, ranging from mild skin and soft tissue infections to severe, life-threatening conditions such as bacteremia, infective endocarditis, necrotizing pneumonia, osteomyelitis, and device-associated infections. The severity of these clinical manifestations is influenced by both bacterial virulence determinants and host susceptibility factors (Cheung et al., 2021; Touaitia et al., 2025).

Accurate and fast identification of *S. aureus* strain characteristics, particularly the detection of resistance determinants such as *mecA*, is critical for guiding effective antimicrobial therapy and implementing infection control strategies. From an epidemiologic view, *S. aureus* colonization is widespread, with approximately one-third of individuals carrying the bacterium at any given time. The incidence of *S. aureus* bacteremia (SAB) in industrialized countries typically ranges from 10 to 30 cases per 100,000 persons annually, with historical case-fatality rates frequently between 10%-30%. MRSA has been a major driver of regional surges in invasive disease and remains associated with increased morbidity, mortality, and prolonged hospitalizations in many healthcare settings (Tong et al., 2015).

Consequently, biosensor-based diagnostic platforms have recently gained attention as innovative alternatives, offering rapid, sensitive, and potentially point-of-care (POC) solutions for detecting bacterial infections and antimicrobial resistance. In this regard, recent years have witnessed remarkable progress in biosensor technologies, which have emerged as powerful alternatives to conventional diagnostics. These devices integrate biological recognition elements, such as antibodies, aptamers, or nucleic acids, with physicochemical transducers that convert biorecognition events into measurable signals. Biosensors can provide rapid, sensitive, and selective detection of pathogens and resistance determinants while remaining compatible with portable and user-friendly formats. Nanomaterial-based optical and electrochemical biosensors, in particular, have achieved outstanding performance, with detection limits down to single-cell levels or femtomolar DNA concentrations, and analysis times under one hour (Gill et al., 2019; Gutiérrez-Santana et al., 2024). The use of gold nanoparticles, graphene derivatives, and hybrid nanocomposites has significantly enhanced surface reactivity, signal amplification, and biocompatibility.

Molecular amplification strategies have also been successfully integrated into biosensor systems. Isothermal amplification techniques such as loop-mediated isothermal amplification (LAMP) and recombinase polymerase amplification (RPA) have replaced thermocycling-dependent PCR, enabling rapid and multiplex detection of genes like *mecA* and *nuc* in simple platforms, including lateral flow and colorimetric biosensors (Sanchini, 2022; Magnano San Lio et al., 2023). Parallel to this, aptamer-based sensors have gained prominence due to their superior stability, cost-effectiveness, and ease of synthesis. When integrated with nanostructured substrates, aptamers enable dual-mode detection readouts, such as fluorescence and surface-enhanced Raman scattering (SERS), that significantly improve diagnostic precision (Chen et al., 2022).

More recently, CRISPR/Cas-enabled biosensing platforms have revolutionized nucleic acid detection. These systems provide single-base specificity and amplification-free signal generation, achieving rapid and selective identification of resistance genes in complex clinical samples (Magnano San Lio et al., 2023; Kao and Alocilja, 2025). Integration with microfluidics and lab-on-a-chip (LOC) technologies further enhances automation, reducing human error and enabling fully contained, sample-to-answer diagnostics in under an hour (Gopal et al., 2022). Such systems demonstrate the tangible potential of biosensor-based antimicrobial resistance (AMR) detection to move beyond proof-of-concept toward real-world application.

**Figure 1:**
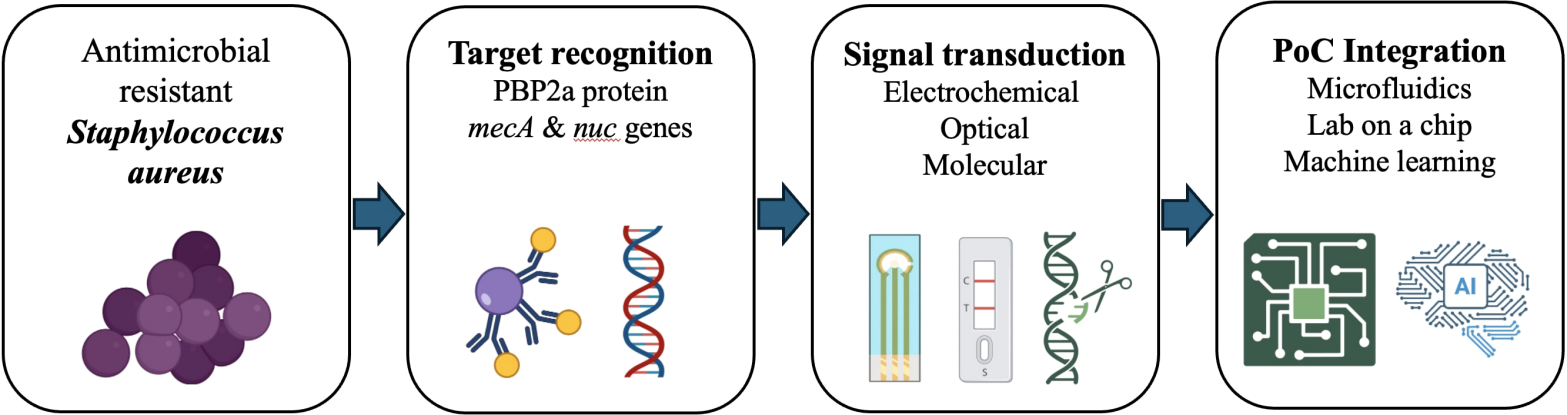
Overview of biosensor-based strategies for antimicrobial resistance detection in *Staphylococcus aureus*. Biosensors employ aptamers, antibodies, and CRISPR-Cas systems to recognize bacterial cells or resistance genes. These interactions generate electrochemical, optical, or molecular signals that could be measured through integrated lab-on-a-chip platforms. These can be coupled with AI-assisted data interpretation, enabling rapid, sensitive, and portable diagnostics for effective antimicrobial stewardship. .

To visually consolidate the main biosensing modalities described above, **Figure 1** summarizes the recognition elements and signal transduction strategies currently employed for the detection of *S. aureus* antimicrobial resistance. This schematic provides a conceptual framework that supports the detailed analysis presented in the following sections.

Nevertheless, several challenges remain. Non-specific adsorption in biological matrices, inconsistent analytical standards, and a scarcity of large-scale clinical validation studies continue to impede clinical translation (Wang and Yang, 2024). To achieve regulatory approval and healthcare integration, these limitations must be addressed through coordinated efforts in standardization, validation, and cost-effective manufacturing.

This review highlights the latest developments and persistent challenges in biosensor technologies for the detection of *S. aureus* antimicrobial resistance, emphasizing how nanotechnology, molecular recognition, and digital integration are converging toward a new generation of rapid, reliable, and field-deployable diagnostic tools.

## 2 Detection of antimicrobial resistant *Staphylococcus aureus*

### 2.1 Surface markers

A primary strategy for *S. aureus* and AMR detection involves targeting the whole bacteria or its surface-exposed resistance markers, such as the PBP2a protein. This approach avoids the need for lysis and can provide a rapid phenotypic assessment. Electrochemical and optical biosensors form the foundation of this strategy, often functionalized with specific recognition elements like antibodies or aptamers to capture the bacteria or target their resistance biomarkers (De Brito Ayres et al., 2022).

Electrochemical biosensors transduce a biological binding event into a measurable electrical signal, such as current, impedance, or potential. A great example is the electrochemical biosensor developed by Li et al. (2023) for the specific detection of MRSA. This sensor targets the whole bacterial cell using a dual-recognition strategy. The detection platform employed a MXene@Au nanocomplex, where MXene (a 2D transition metal carbide) was coupled with AuNPs, creating a high-surface-area conductive substrate for glassy carbon electrode modification. The first target was the D-Ala-D-Ala peptide in the bacterial cell wall, captured by vancomycin-coated magnetic beads. The second is PBP2a protein on the bacterial surface, detected by an immunoglobulin G (IgG)-functionalized electrode. This combination resulted in high specificity, with negligible cross-reactivity against other gram-positive and gram-negative bacteria and achieved a detection limit of 38 CFU/mL in complex clinical samples like cerebrospinal fluid.

Signal amplification strategies are frequently employed to enhance sensitivity. For instance, Xing et al. (2025) developed an electrochemical biosensor integrating a dual-recognition system (anti-PBP2a antibodies and vancomycin) with a MXene-based nanozyme for signal amplification. The nanozyme catalyzed the conversion of a substrate into an electroactive product; this electrochemical activity was monitored using differential pulse voltammetry (DPV) and cyclic voltammetry (CV), enabling the detection of MRSA with a remarkably low LOD of 5.0 CFU/mL and providing precise discrimination between resistant and susceptible strains.

Optical biosensors offer complementary advantages, producing a measurable change in light properties upon target binding. A great example that leverages aptamers is a method which uses DNA aptamers to selectively bind *S. aureus* and guide a silver nanoparticle shell directly onto the bacterial cell. This shell enables the acquisition of a strong Surface-Enhanced Raman Scattering (SERS) fingerprint, which is then analyzed by a convolutional neural network (CNN) to accurately distinguish between methicillin-susceptible (MSSA) and methicillin-resistant (MRSA) strains with 100% accuracy (Wang et al., 2021).

Innovative approaches have also combined different biorecognition elements for sequential detection. Ali et al. (2025) developed a dual-probe biosensor for MRSA that synergizes a capture probe (a PBP2a-specific DNA aptamer on beads) with a detection probe (an RNA-cleaving DNAzyme). The aptamer first enriches MRSA cells by binding the surface protein PBP2a. After lysis, a second internal bacterial protein activates the DNAzyme, which cleaves its substrate to generate a measurable signal, read either by fluorescence or a lateral flow strip. This strategy provided selective MRSA detection over MSSA at 10³ CFU/mL in nasal mucus or serum within one hour.

### 2.2 Genotypic markers

For definitive confirmation of resistance, detection of genotypic markers like the *mecA* or *nuc* genes provides unambiguous results. These methods require a bacterial lysis step and often incorporate nucleic acid amplification to achieve ultra-high sensitivity. This field has been revolutionized by the integration of CRISPR-Cas technology and electrochemical platforms.

CRISPR-Cas enabled platforms are prized for their exceptional specificity for nucleic acid sequences and their compatibility with isothermal amplification methods (e.g., RPA, RAA, LAMP), which are performed at a single temperature and are far more suitable for point-of-care (POC) applications than traditional PCR. The integration of aptamers with CRISPR-Cas technology represents a powerful trend in biosensing, creating hybrid systems that leverage the strengths of both components. This is exemplified by Wei et al. (2022), who designed a colorimetric biosensor where an Ag^+^-binding aptamer served a triple function: as a CRISPR-Cas12a substrate, a colorimetric reaction modulator, and a signal amplifier via C-Ag^+^-C coordination. Coupled with RPA, this system detected MRSA by targeting the *mecA* gene with an exceptional LOD of 8 CFU/mL in clinical samples.

Beyond aptamer partnerships, CRISPR-Cas systems have been fused with a diverse range of isothermal amplification techniques and readout methods. For instance, Xu et al. (2020) integrated rolling circle amplification (RCA) with CRISPR-Cas12a and a fluorescence readout for PBP2a protein detection in culture and blood samples. Similarly, Suea-Ngam et al. (2021) developed a LAMP-CRISPR-Cas12a assay targeting the *mecA* gene with high specificity and an LOD of 3.5 fM in human serum samples.

Electrochemical sensors have also been designed for specific AMR gene analysis with remarkable sensitivity, often using innovative nanomaterial strategies. Dai et al. (2021) fabricated a homogeneous electrochemical DNA sensor for the simultaneous detection of the *mecA* and *nuc* genes. The sensor utilized a metal-organic framework (UiO-66-NH_2_) as a nanocarrier for electroactive dyes, which were released upon target DNA binding. This innovative design achieved detection limits of 3.7 fM for *mecA* and 1.6 fM for *nuc*, demonstrating high accuracy in distinguishing MRSA in real samples. Similarly, Huang et al. (2022) developed a highly sensitive electrochemical biosensor, employing saltatory rolling circle amplification (SRCA) and CRISPR-Cas12a on a gold electrode for the detection of the *nuc* gene. This design used an amperometric mechanism, where target detection prevented silver nanoparticle deposition, leading to a measurable current drop and achieving an LOD of 3 CFUs/mL in spiked milk samples.

A high point of POC innovation is the combination of CRISPR with lateral flow strips (LFS), which provide a simple, instrument-free visual result. For example, Qian et al. (2022) established a portable LFS platform using recombinase-aided amplification (RAA) and CRISPR-Cas12a to detect *S. aureus nuc* gene with an LOD of 1 CFU/mL. Likewise, Wu et al. (2023) used cross-priming amplification (CPA) with CRISPR-Cas12a on an LFS for *mecA* gene, achieving an LOD of 5 CFU/mL in clinical samples.

The biosensor platforms described above vary widely in terms of biorecognition strategy, analytical signal, and achieved sensitivity. To facilitate direct comparison of these methods, **Table 1** summarizes representative examples from the recent literature, highlighting the diverse landscape of biosensors. In practice, a synergistic strategy combining rapid phenotypic screening with confirmatory genotypic analysis optimizes clinical workflow. Emerging integrated designs embody this: for instance, a microfluidic chip that first captures intact bacteria via an anti-PBP2a aptamer and then performs on-chip lysis followed by CRISPR-based detection of *mecA* within a single device. Such systems are invaluable in critical care where comprehensive, rapid information is needed from a single sample.

**Table 1:** Representative biosensor strategies for the detection of antimicrobial resistance in *Staphylococcus aureus*.

Method	Target	Analytical signal	Limit of detection	Key features	Reference
Electrochemical dual-recognition biosensor (MXene@Au + vancomycin-coated magnetic beads + IgG)	D-Ala-D-Ala and PBP2a	Electrochemical Impedance Spectroscopy (EIS)	38 CFU/mL	Dual capture of cell wall and PBP2a.	(Li et al., 2023)
Electrochemical nanozyme-based biosensor (anti-PBP2a + vancomycin, MXene nanozyme)	Whole MRSA cells/PBP2a	Differential pulse voltammetry	5.0 CFU/mL	Distinguishes resistant vs susceptible strains	(Xing et al., 2025)
Dual-probe aptamer + RNA-cleaving DNAzyme hybrid	Surface PBP2a protein + intracellular enzyme	Fluorescence/Lateral flow	~10³ CFU/mL	Dual recognition of PBP2a and internal proteins.	(Ali et al., 2025)
Electrochemical DNA sensor (UiO-66-NH_2_ MOF with electroactive dyes)	*mecA* and *nuc* genes	Differential pulse voltammetry (DPV)	3.7 fM (*mecA*); 1.6 fM (*nuc*)	Simultaneous dual-gene detection	(Dai et al., 2021)
Electrochemical SRCA–CRISPR-Cas12a biosensor	*nuc* gene	Square Wave Voltammetry (SWV)	3 CFU/mL	SRCA-assisted CRISPR assay prevents AgNP deposition on Au electrode	(Huang et al., 2022)
Colorimetric aptamer + CRISPR-Cas12a + RPA hybrid	*mecA* gene	Colorimetric (TMB oxidation)	8 CFU/mL	Aptamer acts as CRISPR substrate, signal modulator, and amplifier	(Wei et al., 2022)
Fluorescent LAMP–CRISPR-Cas12a assay	*mecA* gene	Fluorescence	3.5 fM	Isothermal amplification.	(Suea-Ngam et al., 2021)
RAA–CRISPR-Cas12a lateral flow platform	*nuc* gene	Colorimetric (LFS bands)	1 CFU/mL	Portable, instrument-free point-of-care assay	(Qian et al., 2022)
CPA–CRISPR-Cas12a lateral flow assay	*mecA* gene	Colorimetric (LFS bands)	5 CFU/mL	Portable and Amplification-free.	(Wu et al., 2023)

As illustrated by the strategies summarized in **Table 1**, recent advancements in biosensing for *S. aureus* AMR, from nanomaterial-enhanced electrochemistry to CRISPR-Cas systems, represent a significant leap in detection capability, achieving sensitivities that challenge traditional methods. Their compatibility with miniaturization and simple readouts like lateral flow strips presents a compelling vision for decentralized, point-of-care diagnostics. However, this technological promise is tempered by profound commercialization challenges. The reliance on complex, often single-use nanomaterials and intricate biorecognition elements raises serious concerns regarding reproducible fabrication, cost-effectiveness, and scalable manufacturing, creating a significant gap between laboratory prototypes and viable commercial products.

Also, the suitability of each biosensing modality depends strongly on clinical context. Rapid-throughput environments such as emergency departments or primary care clinics prioritize short turnaround time, minimal training and low per-test cost; here, lateral-flow, colorimetric, and simplified electrochemical platforms offer the most practical balance. In contrast, hospital wards and ICUs can accommodate moderately higher workflow complexity in exchange for greater sensitivity and quantitative capability, making portable electrochemical and microfluidic systems ideal for early therapy adjustment. Reference laboratories and specialized infectious-disease units are best positioned to deploy multiplexed CRISPR- or electrochemical genotyping tools that provide definitive resistance characterization.

This landscape suggests a future of strategic application rather than universal replacement. The operational simplicity, rapid result time, and lower cost of conventional immunodetection will likely ensure its continued relevance for rapid, high-throughput screening where definitive genotypic confirmation is not required. Conversely, the superior specificity of aptamer and CRISPR-based biosensors makes them indispensable for confirmatory testing in critical cases, but their current reliance on lysis and amplification limits their practicality for routine use. Thus, the true challenge lies not only in enhancing sensitivity but in innovating streamlined, integrated, and manufacturable designs that can bridge the gap from sophisticated proof-of-concept to practical clinical tool.

## 3 Microfluidic and integrated lab-on-a-chip platforms

Recent advances in microfluidic and Lab-on-a-Chip (LOC) systems are revolutionizing Antimicrobial Susceptibility Testing (AST) by creating fully integrated, automated platforms. The primary benefit is the dramatic reduction in turnaround time. For example, Lee et al. (2021) developed a system that automates sample and antibiotic preparation for AST in just 10 minutes. Furthermore, contemporary LOC platforms can deliver accurate Minimum Inhibitory Concentration (MIC) results in as little as 2 to 4 hours, a significant improvement over traditional 24–48 hours methods.

A major application of microfluidics is the automation of sample preparation and signal readout. Huang et al. (2019) developed a volumetric bar-chart chip (V-Chip) that provided a visual, quantitative readout for pathogenic bacteria. Their system used aptamer-modified magnetic beads to capture *S. aureus* and platinum nanozymes to catalyze the production of oxygen gas, which pushed an ink bar along a microchannel, the distance traveled correlated with the bacterial concentration, achieving an LOD as low as 1 CFU/mL. Chen et al. (2023) created a portable system featuring a 3D-printed magnetic separation device for sample pretreatment integrated with a lateral flow assay, using nanozymes as colorimetric labels for the detection of *S. aureus*.

A range of sophisticated sensors integrated into these chips enables rapid analysis. Spencer et al. (2020) used impedance sensors to detect bacterial phenotypic responses within a 30-minute incubation window. Similarly, Huang et al. (2020) demonstrated a 2-hour AST using a Surface-Enhanced Raman Scattering (SERS) system. Fabricated with materials like polydimethylsiloxane (PDMS) using modern techniques, these integrated LOC systems represent the forefront of diagnostic technology, poised to deliver rapid, precise AST directly to the point of care and support timely, targeted antibiotic therapy.

The rapid, sensor-integrated microfluidic platforms for AST represent a significant stride toward the overarching goal in analytical chemistry of developing sustainable, low-cost, and easy-to-use methodologies. However, their current incorporation, often reliant on PDMS fabrication and complex electrochemical or spectroscopic sensors, is contradictory with this very objective. Even though sensor-microfluidics tandems can mitigate certain disadvantages, traditional materials such as PDMS are problematic to manufacture, while advanced sensors often imply tradeoffs in terms of either cost, complexity, or the need for specific reagents (Vergara-Barberán et al., 2023). The current commercial platforms, essentially unaffordable, illustrate this translational gap. Thus, the next generation of devices in AST, in order to be fully aligned with the future direction of the field, which is toward paper-based and 3D-printed designs for superior accessibility, needs to focus on these scalable and sustainable alternatives so that the in rapid PoC testing reaches equitably across the world.

## 4 Overview

The rapid evolution of biosensor technologies has profoundly changed the approach to detecting AMR in *Staphylococcus aureus*, yet the path to clinical implementation remains complex. The reviewed literature highlights a broad spectrum of biosensing platforms (electrochemical, optical, aptamer-based, CRISPR-enabled, and microfluidic) that achieve remarkable analytical performance under laboratory conditions. As outlined schematically in **Figure 1**, these platforms rely on distinct recognition mechanisms and transduction modes that can be integrated into increasingly hybrid and automated diagnostic formats.

Among current approaches, platforms that incorporate target-enrichment steps, such as magnetic immunocapture or aptamer-based pull recognition, tend to show the greatest resilience to complex matrices including blood, sputum and wound exudates. Antifouling surface coatings, microfluidic pre-processing, and built-in internal controls further mitigate interference and enhance reproducibility. Progress toward clinical implementation will depend on the development of shared reference materials, multicenter validation cohorts and early regulatory alignment to harmonize analytical reporting and support scalable fabrication.

Electrochemical biosensors have achieved exceptional sensitivity, detecting MRSA at concentrations as low as 1 CFU/mL and sufficient specificity to discriminate resistant from non-resistant serotypes (Ranjbar and Shahrokhian, 2018; Li et al., 2023). Their compatibility with miniaturization and potential for integration into handheld devices make them attractive for point-of-care diagnostics. Nonetheless, issues such as electrode fouling, biofilm formation, and matrix interference significantly reduce performance in real clinical samples (Gill et al., 2019). Optical biosensors, particularly those using colorimetric or SERS-based readouts, provide fast and user-friendly detection but often rely on precise control of reaction conditions (Zhang et al., 2021; Qi et al., 2022).

Aptamer-based biosensors offer a smart solution to several of these challenges by providing high-affinity, cost-effective recognition elements with superior chemical stability (Jin et al., 2017; Cai et al., 2020; Tao et al., 2021). An added advantage is the easy scalability, since they are produced by chemical synthesis. Their adaptability enables hybrid designs that combine fluorescence, Förster resonance energy transfer (FRET), or nanopore sensing with microfluidic platforms, forming the way for multiplex detection of MRSA biomarkers. CRISPR-enabled biosensors add an extra dimension of molecular specificity, allowing the detection of resistance genes such as *mecA* and *nuc* without extensive sample preparation (Xu et al., 2018; Wu et al., 2023). When integrated with isothermal amplification and lateral flow assays, these platforms provide sub-hour, amplification-free detection suitable for field and bedside applications.

Microfluidic and lab-on-a-chip (LOC) technologies are perhaps the most transformative. They integrate multiple steps (sample processing, amplification, and detection) within a single device, minimizing operator dependency and contamination risks (Lee et al., 2021). But, beyond hardware, digital integration is reshaping the biosensor landscape, artificial intelligence and machine learning algorithms are becoming integral to biosensor data processing. They perform critical functions such as cleaning and classifying raw electrochemical or spectral signals, detecting sensor drift, and integrating phenotypic and genotypic data outputs. This allows for the accurate, automated classification of resistant and susceptible strains (Wang et al., 2021). Furthermore, this capability enables a transformative closed-loop “sensor-to-therapy” system. In this model, point-of-care biosensors transmit data to a secure cloud platform. AI models then interpret the complex signals, cross-reference them with regional AMR databases, and return personalized treatment recommendations directly to clinicians.

The next phase of biosensor development must emphasize clinical validation and regulatory alignment. Standardized reporting of analytical metrics, such as limit of detection, reproducibility, and stability, are essential for comparison across technologies (Magnano San Lio et al., 2023). Moreover, multidisciplinary collaboration among different scientific areas will be crucial to develop antifouling strategies, scalable fabrication processes, and affordable platforms.

## 5 Conclusions

Biosensor technologies are rapidly redefining AMR diagnostics in *Staphylococcus aureus*, merging nanotechnology, molecular recognition, and microfluidic automation into compact, high-performance devices. Electrochemical and optical platforms now achieve detection limits as low as 1 CFU/mL, while CRISPR-based assays provide unparalleled molecular specificity without complex instrumentation. The integration of aptamers, nanozymes, and isothermal amplification has further reduced assay time and improved portability, bringing biosensors closer to true point-of-care utility.

Yet, significant challenges persist. Most systems remain at the proof-of-concept stage, lacking large-scale validation, standardized reporting metrics, and robust performance in real biological samples. Future progress will depend on the development of antifouling surfaces, scalable fabrication, and regulatory harmonization to ensure reproducibility and comparability across platforms.

Looking ahead, the convergence of biosensors with artificial intelligence, digital health, and cloud-based data analytics offers a pathway toward continuous AMR surveillance and personalized infection management. By addressing clinical and manufacturing barriers, biosensors could transition from laboratory prototypes to frontline diagnostic tools, delivering faster, smarter, and more accessible detection of MRSA.
